# Systemic Sclerosis Associated Interstitial Lung Disease and Nintedanib: A Rare Disease and a Promising Drug

**DOI:** 10.7759/cureus.16404

**Published:** 2021-07-15

**Authors:** Sunam Kafle, Manusha Thapa Magar, Priyanka Patel, Arisa Poudel, Ivan Cancarevic

**Affiliations:** 1 Internal Medicine, Neurology, California Institute of Behavioral Neurosciences & Psychology, Fairfield, USA; 2 Internal Medicine, California Institute of Behavioral Neurosciences & Psychology, Fairfield, USA

**Keywords:** systemic sclerosis, interstitial lung disease, scleroderma, pulmonary fibrosis, lung fibrosis

## Abstract

Systemic sclerosis-associated interstitial lung disease (SSc-ILD) is a rare disease with a progressive nature, eventually leading to lung fibrosis. Nintedanib, a tyrosine kinase inhibitor, is a widely accepted drug for treating idiopathic pulmonary fibrosis (IPF), a disease that shares some similarities with SSc-ILD regarding pathological disease processes. In this review, we aim to discuss the pathogenesis of SSc-ILD and the overall role of nintedanib in the management of SSc-ILD. SSc-ILD involves multiple pathological mediators contributing to various pathways that ultimately cause lung fibrosis. The pathogenesis of SSc-ILD is a complex phenomenon and still needs further study. Nintedanib has demonstrated its efficacy in the treatment of SSc-ILD by reducing the progression of the pathological process. It has also proven its clinical significance in the management of SSc-ILD. However, the currently available literature does not have any evidence to compare the effectiveness of nintedanib with the already available treatment modalities such as cyclophosphamide (CYC), mycophenolate mofetil (MMF), and azathioprine (AZT). The current literature also lacks information about nintedanib's long-term consequences on patients with SSc-ILD. Therefore, to create better evidence-based treatment guidelines, we recommend that researchers conduct randomized clinical trials comparing nintedanib to MMF, CYC, AZT, etc., and continue surveillance to explore the long-term consequences of nintedanib.

## Introduction and background

Systemic sclerosis (SSc) is a rare connective tissue disease with compound pathogenesis that involves vascular injury, autoimmunity, and unwarranted deposition of extracellular matrix (ECM) leading to progressive fibrosis of various tissues and organs [1,2]. Skin and various internal organs, including lungs, heart, kidneys; gastrointestinal tract; and musculoskeletal system are affected by SSc, giving rise to skin fibrosis and several systemic manifestations [1,2]. The involvement of the lungs causing interstitial lung disease (ILD) is one of the common systemic manifestations of SSc [1,2]. Typically, 50% of SSc patients go on to develop clinically significant ILD, mainly within the first five years from the diagnosis of SSc [1].

At present, ILD is the primary cause of mortality in SSc patients, and it alone accounts for 33% of SSc-related deaths in patients with SSc [3]. Therefore, timely identification of ILD should be done by frequent testing of pulmonary function in these patients by measuring forced vital capacity (FVC) and diffusion lung capacity for carbon monoxide (DLCO) [2]. ILD involves progressive inflammation and fibrosis of lung tissue, and to date, there is no treatment available to reverse lung fibrosis [3]. Therefore, prevention of disease progression by early intervention is the goal of managing this disease [3]. The decline in pulmonary function or radiographic deterioration indicates ILD progression in SSc patients, and such patients should begin treatment regardless of the disease severity [3]. Immunosuppressive therapy with cyclophosphamide (CYC) and mycophenolate mofetil (MMF) is the most widely used treatment modality for SSc-associated ILD [4]. MMF is preferred over CYC due to its better side effect profile and tolerability [4].

Nintedanib is a small molecule designed as an adenosine triphosphate (ATP)-competitive inhibitor of intracellular pro-angiogenic receptor tyrosine kinases [5]. It was initially formulated to treat malignancies such as non-small cell lung cancer and colorectal and ovarian cancers [5]. Later, due to its potential to inhibit processes of fibrotic pathogenesis, it was studied for the treatment of idiopathic pulmonary fibrosis (IPF), where it showed its beneficial effect by reducing the pulmonary function decline, lesser exacerbations, and improved quality of life [6]. IPF shares similarities with SSc-associated ILD regarding pathophysiology leading to pulmonary fibrosis [7]. These diseases require epithelial and/or endothelial cell injury and cell death to initiate the fibrotic cascade [7]. Also, the final common pathway causing lung fibrosis in IPF and SSc-ILD is believed to be the recruitment and activation of myofibroblasts caused by aberrant transformation growth factor-beta (TGF-B) signaling [7]. Myofibroblasts, the specialized fibroblasts, ultimately cause fibrosis in both ILD and IPF by excessive ECM deposition [8].

Nintedanib's widespread use for treating a similar fibrotic condition such as IPF has led to a growing interest in exploring its role in SSc-ILD. Since ILD is one of the most important causes of mortality in patients with SSc, it should have better evidence-based treatment guidelines regarding all the available treatment options. This review aims to discuss the pathways leading to pulmonary fibrosis in SSc-ILD, nintedanib's action on these pathways, and finally, the overall role of nintedanib in the management of SSc-ILD.

## Review

Method

We searched in multiple databases, including PubMed, Scopus, and ScienceDirect, to find relevant studies for our narrative review. The literature search was conducted on April 22, 2021. We used both regular keywords and Medical Subject Headings (MeSH) keywords to search on PubMed. Keywords such as 'systemic sclerosis, scleroderma, interstitial lung disease, pulmonary fibrosis, lung fibrosis, nintedanib, and pathogenesis' were used for the literature search. Boolean operators 'AND' and 'OR' were used to combine the keywords for the literature search. Table 1 shows the keywords in combination for literature search in different databases. We selected 20 articles from the search results which were relevant to our review topic. 

**Table 1:** Keywords in combination for literature search

Keywords in combination	PubMed	ScienceDirect	Scopus
Nintedanib AND Pulmonary fibrosis	684	1141	3860
Nintedanib AND Interstitial lung disease	346	890	2903
Nintedanib AND Systemic sclerosis	79	308	1141
Interstitial lung disease AND Pathogenesis	3933	31432	39408
Scleroderma OR Systemic sclerosis AND Nintedanib	81	354	1145

Discussion

Pathogenesis of Systemic Sclerosis-Associated Interstitial Lung Disease

Pathogenesis of SSc-ILD involves cells of the innate and adaptive immune system, vasculature, and specialized cells such as alveolar epithelial cells (AECs) [9]. The early phase of the disease involves events that may act as a trigger, which could be anything from the lung microbiome to environmental factors such as chemical exposure, recurrent microaspiration, and lung injury [9]. Changes in the lung tissue from these inciting events cause endothelial activation leading to microvascular injury and T and B cell infiltration [8-10]. Microvascular damage also induces inflammation and autoimmunity in the lung tissue, which ultimately stimulates the activation of fibroblasts [8-10]. Many profibrotic cytokines, chemokines, and pro-inflammatory mediators such TGF-B, platelet-derived growth factor (PDGF), and vascular endothelial growth factor (VEGF) have been implicated to have a role in the pathogenesis of SSc-ILD through the activation of lung fibroblasts [8-11].

**Figure 1:**
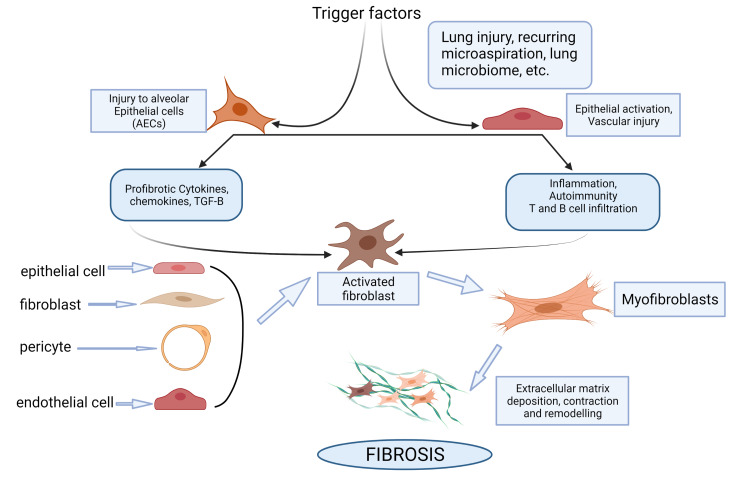
Pathogenesis of lung fibrosis in systemic sclerosis-associated interstitial lung disease TGF-B: transformation growth factor-beta

Fibroblasts involved in lung fibrosis are considered a distinct subgroup of cells with origins from various cell lineages, including locally activated fibroblasts, epithelial cells, endothelial cells, and pericytes [8-10]. Activated and specialized forms of these fibroblasts, known as myofibroblasts, have both synthesizing features like fibroblasts and contractile properties like smooth muscle cells (SMC) [8]. Alpha-smooth muscle actin (a-SMA) stress fibers contribute to the contractility of myofibroblasts, and endothelin-1 (ET-1), a cytokine, has been shown to induce expression of a-SMA in these cells [12]. Studies have reported an increased expression of ET-1 and ET-1 receptors on interstitial vessels of patients with SSc-ILD [12]. Bronchoalveolar lavage (BAL) fluid acquired from patients with SSc has been cultured, and it has shown fibronectin, excessive collagen production, and spontaneous growth of cells with a-SMA [13]. These findings are not seen in the BAL fluid culture of healthy people, which implies that myofibroblasts can produce ECM even outside the fibrotic area [13]. Myofibroblasts ultimately cause fibrosis by excessive deposition of ECM, remodeling, and contraction of tissues [8]. The pathways leading up to lung fibrosis are summarized in Figure 1. Progressive fibrosis of the lung parenchyma and interstitial tissue ultimately results in mechanical stiffness and altered lung function in patients with SSc-ILD [8-11].

SSc-ILD is a rare disease involving multiple cell populations and different pathogenetic mechanisms ultimately contributing to lung fibrosis. Although the general idea regarding pathogenesis is known, the primary pathway, the crucial steps, and the most crucial mediator are still complex to define. Therefore, a definitive mechanism behind the pathogenesis is yet to be explored. Clarity regarding the pathogenesis will help develop new drugs that could work more effectively by targeting the specific pathways. A clearer understanding of pathogenesis will eventually help build advanced treatment modalities, stop the disease progression, and better manage the disease. More studies on molecular pathogenesis, cellular model of disease, and epigenetic research are required to have a broader and more detailed picture of SSc-ILD pathogenesis.

Nintedanib in Pulmonary Fibrosis

Nintedanib, an intracellular tyrosine kinase inhibitor, has been shown to inhibit tyrosine phosphorylation of type 2 TGF-B receptor, decrease synthesis of ECM protein and a-SMA by down-regulating the messenger-RNA expression [5,12,14]. In a study by Atanelishvili et al., lung fibroblasts were stimulated by PDGF in a cellular model of SSc-ILD in the presence and absence of nintedanib [12]. Cells stimulated in the presence of nintedanib demonstrated a decrease in the production of collagen and fibronectin, and a reduction in the proliferation rate by 1.9-fold within 24 hours, compared to the other group of cells [12]. This study suggested that nintedanib has the potential to prevent as well as reverse the increased activity of lung fibrosis [12]. Huang et al. conducted a study on murine sclerodermatous chronic graft-versus-host disease model and tight-skin-1 mice, where nintedanib was given in tolerated dose to these mice [14]. Nintedanib improved fibrosis in these mice models by inhibiting different cytokines such as TGF-B and PDGF, the essential mediators in activating lung fibroblasts [14]. Nintedanib has also shown its immunomodulatory effects by blocking T-cell stimulation [15].

Nintedanib has proven to be a promising drug for SSc-ILD by inhibiting the process of lung fibrosis. It has demonstrated its anti-fibrotic and anti-inflammatory role in various pathological processes leading to lung fibrosis. Since SSc-ILD is a progressive disease involving multiple phases, studying the effect of nintedanib in various stages of the disease process would provide more in-depth insight into the drug's mechanism of action in this disease. We recommend that researchers conduct more studies on animal and cellular models regarding nintedanib's action on SSc-ILD.

Clinical Efficacy of Nintedanib in Systemic Sclerosis-Associated Interstitial Lung Disease

The first literature demonstrating the clinical benefit of nintedanib in a human subject with SSc-ILD was a case report published in 2018 by Duarte et al. [16]. A 65-year-old female was diagnosed with SSc at age 41 and later developed ILD at the age of 48 years [16]. Despite being on multiple immunosuppressants, the patient developed progressive lung fibrosis and had worsening symptoms [16]. Therefore, the patient was suggested to initiate nintedanib 150 mg twice daily [16]. At the end of a year of treatment with nintedanib, the patient's health was improved clinically, FVC was slightly increased, and the necessity for supplemental oxygen therapy was decreased [16].

The Safety and Efficacy of Nintedanib in Systemic Sclerosis (SENSCIS) trial, a double-blinded, large-scale, randomized clinical trial (RCT), was conducted to compare nintedanib's effectiveness and safety profile compared to placebo in patients with SSc-ILD [17]. The patients were recruited from 32 different countries [17]. A total of 576 patients were included in the study and were divided into two groups: 288 receiving nintedanib and the other 288 receiving placebo [17]. The study subjects included patients older than 18 years, who had SSc diagnosed by the established criteria. ILD was identified by a high-resolution computed tomographic (HRCT) scan showing lung fibrosis affecting a minimum of 10% of the lungs [17]. Patients with severe progressive ILD whose FVC was less than 40% and DLCO less than 30% of the predicted value were excluded from the study [17]. The primary end-point of the trial was the yearly decline rate of FVC, which was assessed over 52 weeks [17]. Of the total patients participating in the trial, 232 from the nintedanib group and 257 from the placebo group completed the 52-week intervention [17]. At the end of 52 weeks, the primary end-point analysis was done, which showed that the annual rate of decline of FVC was 52.4 ml in patients receiving nintedanib and 93.3 ml in those receiving placebo [17]. There was a difference of 41.0 ml per year in the decline rate of FVC between these two groups with a 95% confidence interval, 2.9 ml to 79.0 ml, and a p-value of 0.04, which demonstrated that the findings were statistically significant [17].

Multiple subgroup analyses were done from the SENSCIS trial and published by different authors [18,19]. Subgroup analysis of Asian and non-Asian patients was done by Azuma et al. [18]. Out of the total 576 patients from the trial, 62 Asian patients were enrolled in the nintedanib group and 81 patients in the placebo group [18]. The analysis showed that the annual rate of FVC decline was similar in both Asian (99.9 ml) and non-Asian (90.6 ml) patients receiving placebo [18]. Likewise, the annual rate of FVC decline in the other group receiving nintedanib was also consistent between Asian (44.3 ml) and non-Asian (39.0 ml) patients [18]. Another subgroup analysis was done between patients receiving MMF at baseline and those not receiving MMF [19]. In the group of patients receiving MMF at baseline, the annual rate of FVC decline was 40.2 ml in patients receiving nintedanib and 66.5 ml in patients receiving placebo [19]. In the other group of patients not receiving MMF at baseline, the annual rate of FVC decline was 63.9 ml in patients receiving nintedanib and 119.3 ml in patients receiving placebo [19]. This subgroup analysis demonstrated that the effect of nintedanib was numerically higher in the group where the patients were not taking MMF [19].

A case series published by Martinez et al. studied the effect of nintedanib in four patients with severe, progressive fibrotic SSc-ILD who were undergoing evaluation for lung transplant [20]. The SENSCIS trial had excluded patients with severe lung fibrosis with FVC less than 40% and DLCO less than 30% of the predicted value, those with severe pulmonary hypertension, and those awaiting lung transplant [20]. Despite their severe disease, these four patients were started on nintedanib, and the functional decline of their pulmonary function under nintedanib was evaluated [20]. The descriptive data obtained from these patients showed that two out of four patients had significant improvement in FVC, and the other two had a decrease in the decline of FVC and DLCO after initiation of nintedanib [20].

The SENSCIS is the only RCT study to date that has assessed nintedanib's efficacy in patients with SSc-ILD [17]. The effectiveness of nintedanib in the management of SSc-ILD is evident from these studies [16,17,20], making it an FDA-approved drug to treat this disease [21]. It decreases disease progression by various mechanisms and ultimately helps in the improvement of the health condition of patients suffering from SSc-ILD. However, we need more evidence-based studies to support the efficacy of this drug on SSc-ILD. One single clinical trial and a couple of case reports shouldn't be considered sufficient evidence in this modern era of medicine.

Recommendation

In summary, the role of nintedanib is proven to be beneficial in the management of SSc-ILD. The SENSCIS trial had a follow-up period of 52 weeks, and the decrease in the decline rate of FVC of the patients was the only clinical outcome documented at the end of the trial [17-19]. Long-term monitoring and surveillance regarding the effects of this drug on patients with SSc-ILD are lacking. The other possible clinical outcomes of the drug on patients with SSc-ILD need to be explored. We could not find any study comparing its efficacy with the widely used medications for SSc-ILD such as MMF, azathioprine (AZT), and CYC from literature research. It is crucial to compare this newly approved drug to the other immunosuppressants commonly used to manage this disease. We also have only a few case reports showing the effect of this drug in patients with SSc-ILD. The ultimate goal in this era of medicine is to explore all the possible domains of evidence-based management to address the patient's condition in the best way possible. Therefore, we suggest that researchers conduct more RCTs comparing the efficacy of nintedanib to placebo and other widely used drugs for SSc-ILD. Given the extremely rare nature of the disease and challenges in running a large-scale RCT, clinicians are encouraged to publish reports on the unique cases of SSc-ILD treated with nintedanib. These individual reports with significant and distinct clinical outcomes could potentially add to the evidence of nintedanib's role in SSc-ILD treatment and ultimately help develop a better evidence-based algorithm for managing patients with SSc-ILD.

Limitation

We could not access the full text of few articles that seemed relevant while going through their titles and abstracts; hence, some important studies might have been missed, obscuring our review's overall discussion and conclusion. We have not discussed the side effect profile of nintedanib, which is also a potential factor influencing its role in the general management of SSc-ILD patients.

## Conclusions

Our review has discussed and analyzed the pathogenesis of SSc-ILD and the role of nintedanib in the treatment of SSc-ILD. SSc-ILD is a complex disease with a chronic course, multiple pathways of pathogenesis, and progressive nature, ultimately leading to lung fibrosis. Nintedanib has shown to inhibit multiple mediators and pathways of lung fibrosis, ultimately reducing the progression of ILD in SSc patients. It has proven to be a promising drug in the overall clinical improvement and management of SSc-ILD. Our suggestion to the researchers and clinicians is to conduct more large-scale RCTs, publish multiple case reports with unique clinical outcomes in SSc-ILD patients treated with nintedanib, and continue long-term post-marketing surveillance of the drug. We believe that these studies will help develop more advanced evidence-based management guidelines for patients living with SSc-ILD and improve their overall quality of life.
